# Effects of supplementing diet with *Lactiplantibacillus plantarum* LP100 on growth, gut health, immunity, and disease resistance in *Scatophagus argus*

**DOI:** 10.3389/fmicb.2026.1862630

**Published:** 2026-07-07

**Authors:** Ruirui Zhang, Yiming Deng, Jingbin Feng, Suxiao Jia, Qingying Li, Haixu Ou, Haidong Gao, Liying Wang, Zhihong Lin, Youhou Xu

**Affiliations:** 1 College of Animal Science & Technology, Guangxi University, Nanning, China; 2 Guangxi Key Laboratory of Marine Environmental Disaster Processes and Ecological Protection Technology, Beibu Gulf Marine Ecological Environment Field Observation and Research Station of Guangxi, College of Marine Sciences, Beibu Gulf University, Qinzhou, China

**Keywords:** growth performance, gut microbiota, immune function, *Lactiplantibacillus plantarum* LP100, *Scatophagus argus*

## Abstract

The application of *Lactiplantibacillus plantarum* LP100 in *Scatophagus argus,* including its effective dietary, remains unclear. This study evaluated the effects of dietary LP100 supplementation at 1.1 × 10^6^, 1.1 × 10^7^, 1.1 × 10^8^, and 1.1 × 10^9^ CFU/g to identify a suitable inclusion level for *S. argus*. A total of 300 fish were randomly assigned to five groups, including a control group, with three replicate tanks per group and 20 fish per tank. After the 8-week feeding trial, LP100 supplementation at appropriate concentrations significantly increased weight gain rate and specific growth rate, reduced feed conversion ratio, increased serum lysozyme activity, complement C3 and C4 levels. Compared with the CON group, all LP100-supplemented groups had significantly higher hepatic SOD activity (highest in LPMH, *p* < 0.05), while the LPMH and LPH groups exhibited significantly lower hepatic MDA content (*p* < 0.05). LP100 supplementation was also associated with increased intestinal expression of immune-related genes, including the pro-inflammatory cytokine *IL-1β* and *TNF-α*, the anti-inflammatory cytokine *IL-10*, and signaling molecules *IRAK-4*, *MyD88*, and *TLR2*. Intestinal histomorphology showed an improving trend, with increased villus length, villus width, and muscularis thickness. Microbial community analysis showed that Firmicutes and Proteobacteria were the dominant phyla across all groups. The LPMH group (1.1 × 10^8^ CFU/g) showed a distinct microbial profile, with higher Firmicutes abundance than the control group (53.3% vs. 25.3%) and lower Bacteroidota abundance (0.18% vs. 0.67%). Opportunistic pathogenic bacteria decreased, whereas probiotic genera, including *Bacillus* and *Lysinibacillus*, increased in abundance. After *Streptococcus agalactiae* challenge, the survival rate was significantly higher in the LPMH group than in the control group (60% vs. 16.67%; *p* < 0.05). Under the present experimental conditions, 1.1 × 10^8^ CFU/g may represent a suitable dietary supplementation level for LP100 in *S. argus.*

## 1 Introduction

According to the FAO, aquatic animal foods provide approximately 15% of animal protein and 6% of total protein consumed globally, supporting their role in global nutrition and food security (FAO, 2024). Aquaculture production has expanded rapidly over recent decades and now supplies more than half of the fish consumed worldwide (Liu R. et al., 2024). However, expanded production has increased the complexity of disease prevention and control. According to the *2024 Aquatic Animal Health in China*, disease-related economic losses in China’s aquaculture industry reached RMB 18.76 billion in 2023, with bacterial diseases accounting for approximately 52.3% of total losses (Bureau of Fisheries, Ministry of Agriculture and Rural Affairs and National Fisheries Technology Extension Center, 2024). Therefore, safe, efficient, and environmentally sustainable disease-control strategies are needed to support the continued development of aquaculture.

*Streptococcus agalactiae* (Group B Streptococcus, GBS) is a gram-positive opportunistic pathogen that causes severe disease in multiple cultured fish species. Infection can result in septicemia, meningitis, and multi-organ damage. Infected fish may show abnormal swimming, loss of equilibrium, exophthalmos, cutaneous and fin-base hemorrhage, hepatosplenomegaly, and ascites (Su et al., 2019; Maniyappan et al., 2025). Although *S. agalactiae* has been studied mainly in freshwater aquaculture, infections have increasingly been reported in marine and brackish-water fish with the expansion of coastal aquaculture (Gao et al., 2023). This has increased concern regarding its potential impact on emerging euryhaline aquaculture species.

*Scatophagus argus*, commonly known as the spotted scat, belongs to the family Scatophagidae and genus *Scatophagus* (Wang, 2022). It is widely distributed in the Indo-Pacific region and mainly inhabits estuaries, coastal waters, mangroves, and coral reef-associated areas. As a euryhaline fish, *S. argus* tolerates salinity fluctuations and has attracted increasing attention as a promising aquaculture species. Its culture has expanded in the southern coastal regions of China due to its economic value and environmental adaptability (Su et al., 2022). Fish health and production performance are closely related to digestive capacity, immune status, and intestinal microbial balance. In teleosts, the intestine is the major site of nutrient digestion and absorption and an important mucosal immune tissue involved in epithelial barrier function, host defense, and microbial regulation. The gut microbiota also contributes to nutrient metabolism, immune modulation, intestinal homeostasis, and resistance to pathogen colonization. Under intensive aquaculture conditions, environmental fluctuations and handling stress may increase the susceptibility of cultured *S. argus* to bacterial pathogens, reducing survival and economic returns (Lu et al., 2022). However, information on *S. agalactiae* infection in *S. argus* and other marine or estuarine fish species remains limited. Because species-specific epidemiological data remain limited, *S. agalactiae* was selected in this study as a representative Gram-positive bacterial pathogen for immune challenge rather than a confirmed major pathogen of *S. argus* aquaculture. This limitation also supports the need for targeted, environmentally sustainable disease-control strategies for emerging marine aquaculture species.

Antibiotics remain a common strategy for controlling bacterial diseases in aquaculture. However, misuse and prolonged use can select for drug-resistant strains and lead to pharmaceutical residues, posing risks to aquatic food safety and the surrounding ecosystem (Maniyappan et al., 2025). Antibiotics and antibiotic resistance genes (ARGs) have also been detected in coastal waters (Adenaya et al., 2023), likely reflecting intensive antibiotic use in aquaculture, aquaculture effluent discharge, and riverine inputs (Hermansson et al., 1987). Therefore, non-antibiotic disease-control strategies are needed. Probiotics are used as functional feed additives and can improve fish health by regulating intestinal microbiota, immune responses, and growth performance (Nadella et al., 2021).

Several probiotic taxa, including *Lactiplantibacillus plantarum*, *Clostridium butyricum*, *Lactobacillus acidophilus*, and *Bacillus subtilis*, have been evaluated in aquaculture species (He et al., 2024; Pan et al., 2023; Setufe et al., 2025; Shahraki et al., 2025). *L. plantarum* is a gram-positive, rod-shaped, non-spore-forming, aerotolerant anaerobe with an optimal growth temperature of 30–35 °C (Cao et al., 2018). In aquaculture, dietary *L. plantarum* have been reported to improve growth, feed utilization, immune function, and health status in fish and shrimp (Setufe et al., 2025; Zhang et al., 2025a). Beneficial effects have been reported in bighead catfish (*Clarias macrocephalus*), coho salmon (*Oncorhynchus kisutch*), and Nile tilapia (*Oreochromis niloticus*) (Hien et al., 2021; Zhang et al., 2025b; Cheng et al., 2025).

The effects of probiotics depend on the strain, host species, and culture environment. Different *L. plantarum* strains differ in exopolysaccharide (EPS) production, gastrointestinal stress tolerance, immunomodulatory activity, pathogen inhibition, and environmental stress responses. For example, variation in EPS production can affect acid and bile salt tolerance, cell surface properties, and TLR2-mediated immune signaling (Lee et al., 2016). Strain-dependent protection against *Salmonella* infection has also been reported, indicating that not all *L. plantarum* strains have equivalent pathogen-inhibitory activity (Liu et al., 2018). In addition, different strains show distinct metabolic responses and tolerance mechanisms under cadmium exposure (Zhai et al., 2018). These findings indicate that strain-specificity is required before a probiotic is applied to a target host or aquaculture system.

LP100 is a strain of *L. plantarum* isolated from the intestinal tract of maricultured grouper. Unlike strains derived from terrestrial animals, fermented foods, or freshwater fish, LP100 originates from the gastrointestinal environment of a marine fish. This origin may make LP100 better suited to marine or euryhaline fish and to culture conditions involving salinity fluctuation (Han et al., 2025). However, the effects of LP100 in *S. argus* and its appropriate dietary inclusion level have not been established. Therefore, this study evaluated the effects of dietary LP100 supplementation at different concentrations on growth performance, survival, immune function, antioxidant status, intestinal morphology, gut microbiota, and resistance to *S. agalactiae* challenge in *S. argus*.

## 2 Materials and methods

All animal experiments were conducted in accordance with the Guidelines for the Care and Use of Laboratory Animals. The experimental design and procedures were approved by the Animal Ethics Committee of Beibu Gulf University (Approval No. YXDW089). To reduce observer bias, tissue staining, morphological assessment, qRT-PCR analysis, and microbial community sequencing were performed by researchers who were blinded to the treatment groups.

### 2.1 Experimental diets and design

The main components of the basal diet are listed in Table 1, in accordance with the international standard GB/T 22919.9—2024. *L. plantarum* LP100, used in this study, was preserved at the Guangdong Microbial Culture Collection Center (GDMCC68358). The *S. agalactiae* strain TOS01 was provided by Professor Cai Xiaohui from Beibu Gulf University.

**Table 1:** Formulation and proximate composition.

Raw material	Test formulation (absolute dry weight, %)
Fish meal	30.0
Chicken powder	14.0
Soybean meal	16.0
Rapeseed meal	2.00
Peanut meal	10.0
Wheat flour	8.00
Bran	3.40
Choline chloride	0.200
CaH_2_PO_4_	2.00
Lys	0.20
Met	0.20
Tapioca starch	2.00
Vitamin and mineral premix^a^	1.00
Bentonite	2.00
Cr_2_O_3_	1.00
Fish oil	8.00
Total	100.0
Nutrient level (%)	
Crude protein	43.93
Crude lipids	11.85
Ash	11.04

^a^Vitamin A, 350,000 IU; vitamin D₃, 110,000 IU; vitamin E, 4,000 mg; vitamin K₃, 300 mg; vitamin B₁, 400 mg; vitamin B₂, 1,500 mg; vitamin B₆, 600 mg; vitamin B₁₂, 3.5 mg; vitamin C, 6,300 mg; D-calcium pantothenate, 2,000 mg; niacin, 2,500 mg; folic acid, 250 mg; D-biotin, 5.0 mg; inositol, 3,500 mg; magnesium, 2,000 mg; zinc, 2,250 mg; manganese, 1,300 mg; copper, 400 mg; iron, 6,800 mg; cobalt, 120 mg; iodine, 120 mg; selenium, 25 mg.

For LP100 preparation, *L. plantarum* was inoculated into both MRS broth and cultured at 35 °C with shaking at 150 rpm for 12 h. Cells were collected by centrifugation at 4,000 × *g* for 5 min. The supernatant was removed, and the bacterial pellet was washed three times with sterile phosphate-buffered saline (PBS 1M, pH 7.2). The pellet was then resuspended in an appropriate volume of PBS and homogenized. The required volume of the bacterial suspension was evenly sprayed onto the dry feed, then gently mixed to ensure uniform probiotic distribution. The volume and concentration of the suspension were adjusted to obtain final LP100 concentrations of 1.1 × 10^6^, 1.1 × 10^7^, 1.1 × 10^8^, and 1.1 × 10^9^ CFU/g feed.

The probiotic-sprayed diets were air-dried at room temperature 15 °C. The initial spraying concentration was adjusted based on the post-drying survival rate (68–82%) to achieve the target viable counts in the final feed. The diets were then sealed in sterile bags and stored at 4 °C until use. During the feeding trial, only the amount of feed required for each feeding was removed to avoid repeated temperature changes. The viable count of LP100 in the probiotic diets was determined using MRS agar plate counting before feeding to confirm that the target supplementation levels had been achieved. The basal feed was stored at −20 °C before probiotic supplementation (Mohammadian et al., 2022).

The experiment included five treatment groups: a control group (CON) fed the basal diet without probiotic supplementation and four experimental groups fed diets supplemented with LP100 at 1.1 × 10^6^ CFU/g (LPL), 1.1 × 10^7^ CFU/g (LPLH), 1.1 × 10^8^ CFU/g (LPMH), and 1.1 × 10^9^ CFU/g (LPH). These dose levels were selected to cover the commonly used effective range of *L. plantarum* and other lactic acid bacteria in fish feeding trials and to provide preliminary dose–response information for LP100 supplementation in *S. argus* (Pourgholam et al., 2016; Zhang et al., 2025b).

### 2.2 Feeding trials and sample collection

A total of 550 healthy *S. argus* juveniles of similar size were obtained from a commercial hatchery in Beihai City, Guangxi Province, and acclimatized in the laboratory for 2 weeks. During acclimation, 50% of the seawater was replaced daily. Before the feeding trial, fish were fasted for 24 h. A total of 300 fish were then randomly chosen and assigned to 15 culture tanks, with 20 fish per tank. The overall mean initial body weight across groups was 20.09 ± 0.15 g.

The 15 tanks were randomly assigned to five treatment groups, with three replicate tanks per group. Fish were fed twice daily at 08:00 and 18:00. At the beginning of the trial, feed was provided at 2% of body weight per feeding, and the feeding amount was adjusted according to fish growth during the 8-week trial. Feeding behavior was observed during each feeding. Uneaten food was collected, rinsed, dried, and weighed to calculate actual feed intake per tank. Each day, 40% of the water was renewed, and water temperature, feeding behavior, mortality, and individual body weight were recorded. Throughout the trial, water temperature was maintained at 29 ± 1 °C, dissolved oxygen was maintained above 5 mg/L, pH ranged from 7.8 to 8.0, and ammonia concentration was kept below 0.05 mg/L.

After the feeding trial, fish were fasted for 24 h. The total weight and number of fish in each tank were then recorded. Three fish from each tank were randomly selected and anesthetized with 80 mg/L MS-222, and total length and body weight were recorded. Blood samples (1.5 mL) were collected from the caudal veins. After clotting at room temperature for 40 min, samples were centrifuged at 4,000 × g for 10 min at 4 °C. Serum was carefully, visually inspected for hemolysis, aliquoted, and stored at −80 °C until analysis.

For tissue collection, three fish from each tank were randomly selected and immersed in 350 mg/L sodium bicarbonate-buffered MS-222 solution for euthanasia. A 1-cm segment of mid-intestine was rinsed with sterile physiological saline and fixed in 10% neutral buffered formalin for histological analysis (Chen L. et al., 2025). Intestinal contents were gently extruded into sterile cryovials, snap-frozen in liquid nitrogen, and stored at −80 °C for gut microbiota analysis. Intestinal tissues were rinsed with sterile saline, placed into sterile cryovials, snap-frozen in liquid nitrogen, and stored at −80 °C for gene expression analysis. Liver tissues were rinsed with sterile saline, placed into sterile cryovials, snap-frozen in liquid nitrogen, and immediately stored at −80 °C for biochemical analysis.

### 2.3 Growth performance indicators

Before and after the feeding trial, fish were weighed individually. Daily feed intake and mortality were recorded for each tank throughout the trial. Weight gain ratio (WGR), specific growth rate (SGR), feed conversion ratio (FCR), and survival rate (SR) were calculated as follows:


$$\mathrm{WGR}\;(\%)=\frac{\text{Final body weight}-\text{Initial body weight}}{\text{Initial body weight}}\times 100\%$$

$$\mathrm{SGR}\;(\%)=\frac{\ln(\text{Final body weight})-(\text{Initial body weight})}{\text{duration of rearing period}}\times 100\%$$

$$\mathrm{FCR}\;(\%)=\frac{\mathrm{Dry}\;\text{feed intake}}{\text{Final body weight}-\text{Initial body weight}}\times 100\%$$

$$\mathrm{SR}\;(\%)=\frac{\text{Initial number of fish}}{\text{Final number of fish}}\times 100\%$$

### 2.4 Serum biochemical markers and liver antioxidant markers

Serum alkaline phosphatase (ALP) activity was measured using 0.015 mL of serum per sample according to the assay kit from the Nanjing Jiancheng Bioengineering Institute. Complement C3, complement C4, and lysozyme (LZM) were measured using 0.054 mL of serum per sample and commercial ELISA kits from Shanghai Hepeng Biotechnology Co., Ltd.

For liver antioxidant analysis, approximately 0.1 g of liver tissue was placed in a 1.5-mL sterile, enzyme-free centrifuge tube containing 1 mL of extraction solution and grinding beads. Samples were homogenized at low temperature and centrifuged at 12,000 × *g* for 10 min at 4 °C. The supernatant was collected for analysis. Superoxide dismutase (SOD) activity and malondialdehyde (MDA) content were measured according to the instructions of the SOD and MDA assay kits from Suzhou Grace Bio-Reagent Co., Ltd.

### 2.5 Intestinal morphology

For intestinal morphology analysis, a 1-cm segment of the mid-intestine was collected from three *S. argus* per tank, with nine fish analyzed per treatment group. The intestinal lumen was gently rinsed with physiological saline to remove residual contents, and the tissue was fixed in 10% neutral buffered formalin. Samples were dehydrated, embedded in paraffin, sectioned, and stained with hematoxylin and eosin (H&E). Sections were examined using a light microscope, and villus length, villus width, and muscularis thickness were measured. The sample size was selected based on commonly used ranges in similar studies of fish intestinal morphology and the 3Rs principle of animal ethics; however, this sample size may limit the detection of small morphological differences.

### 2.6 Gut microbiota analysis

Gut microbial DNA was extracted using the HiPure Soil DNA Extraction Kit (Magen, Guangzhou, China) according to the manufacturer’s instructions. After 24 h of fasting, sufficient intestinal contents remained for microbial DNA extraction, and samples that met DNA quality and library-preparation requirements were used for sequencing. The V3–V4 region of the bacterial 16S rRNA gene was amplified by PCR using primers 341F (5′-CCTACGGGNGGCWGCAG-3′) and 806R (5′-GGACTACHVGGGTATCTAAT-3′). Amplicon library construction and sequencing were performed by GDIOD Bio Co., Ltd. (Guangdong, China).

Raw reads were filtered with fastp^1^ to obtain clean reads, then merged with FLASH (v1.2.11). OTU clustering at 97% similarity and chimera removal were performed using USEARCH (v11.0.667). Taxonomic annotation was performed using the RDP Classifier (v2.14) against the SILVA database (v138.2). Alpha-diversity indices were calculated in R. Beta diversity was assessed using principal coordinate analysis (PCoA), non-metric multidimensional scaling (NMDS), and permutational multivariate analysis of variance (PERMANOVA) based on Bray-Curtis, Jaccard, and UniFrac distance matrices with the vegan package (Yang et al., 2022).

### 2.7 Quantitative real-time PCR analysis of intestinal immune-related genes

Total RNA was extracted from intestinal tissues using the RNAiso Plus Kit (Takara Bio Inc., Dalian, China). Before reverse transcription, genomic DNA contamination was eliminated using the gDNA Eraser included in the PrimeScript™ RT Reagent Kit with gDNA Eraser (Takara, Japan). Complementary DNA (cDNA) was synthesized according to the manufacturer’s protocol. Real-time quantitative PCR was performed using the Bio-Rad Real-Time Fluorescent Quantitative PCR System (Bio-Rad Laboratories, Inc., Hercules, CA, USA).

Each reaction was performed in a final volume of 20 μL containing 10 μL of TB Green Premix Ex Taq II (Dalian Bao Bioengineering Co., Ltd., Dalian, China), 0.3 μL of each specific primer, 1 μL of template cDNA, and 8.4 μL of enzyme-free deionized water. The thermal cycling was as follows: initial denaturation at 95 °C for 1 min; 40 cycles of 95 °C for 10 s and 60 °C for 30 s; and dissociation analysis at 95 °C for 10 s, 60 °C for 5 s, and 95 °C for 5 s. A no-template control (NTC), in which RNase-free water was used instead of cDNA, and a no-reverse transcriptase control (NRT) in which non-reverse-transcribed RNA was used, were included to detect reagent contamination and genomic DNA contamination. After amplification, melting curve analysis was performed over the range 75–95 °C to confirm amplification specificity. Each reaction was run in triplicate. Primers used for the target genes are listed in Table 2. Relative gene expression was calculated using the 2^−ΔΔCt^ method (Livak and Schmittgen, 2001), with β-actin used as the endogenous reference gene.

**Table 2:** qRT-PCR primer sequences.

Gene	Primer sequence (5′-3′)	Product size (bp)	GenBank
Housekeeping internal reference gene (*β-actin*)	F: GAGAGGTTCCGTTGCCCAGAG	144	KF649214.1
	R: CAGACAGCACAGTGTTGGCGT		
Toll-like receptor 2 (*TLR2*)	F: GCGAAGAAGAAGCCAAAGTT	82	124,075,138
	R: CCCAACCAGAGTCCATTTCA		
Myeloid differentiation primary response 88 (*MyD88*)	F: GCCTGTGACTTTCAGACCAAGT	128	124,049,709
	R: CATAAGGTGAGGAAGCGTAAGA		
Interleukin-1 receptor-associated kinase 4 (*IRAK-4*)	F: GAGAGGAAAAACAGACGACCAG	80	124,061,859
	R: AAATCCAGTGAAATGCTCTTGA		
Tumor necrosis factor-α (*TNF-α*)	F: GGGGACAGACTGAAGACAGAGA	86	124,075,138
	R: AGGCAAACACACCAAAGAAAGT		
Interleukin-1β (*IL-1β*)	F: CAGTTACAAACGATCAGCGACT	114	124,051,293
	R: GATGAACCAACCACAGCACTTA		
Interleukin-10 (*IL-10*)	F: GTGACAAACGACATCAGGGACT	84	124,056,583
	R: ATTGGTGACATCAGTGTTGAGC		

### 2.8 *Streptococcus agalactiae* challenge experiment

At the end of the 8-week feeding trial, 10 fish were randomly selected from each replicate tank within each treatment group for the challenge test, yielding 30 fish per treatment. The challenge test included three replicates, with 10 fish per replicate. Fish were fasted for 24 h before challenge.

*S. agalactiae* was cultured in BHI broth at 37 °C for 8 h. The culture was centrifuged at 5,000 × *g* for 10 min at 4 °C, and the bacterial pellet was collected. The pellet was washed three times with sterile physiological saline, resuspended, and adjusted to a final concentration of 1.5 × 10^8^ CFU/mL. This concentration was selected based on a preliminary dose-mortality trial and resulted in approximately 80% cumulative mortality among control fish fed the basal diet. The same trial also confirmed the safety of the 0.25 mL injection volume, as saline-injected controls showed no mortality or abnormal behavior.

The challenge was performed by intraperitoneal injection. Before injection, fish were anesthetized with 80 mg/L MS-222. Each fish was intraperitoneally injected (Li et al., 2024) with 0.25 mL of the bacterial suspension, corresponding to 3.75 × 10^7^ CFU/fish. After the challenge, fish were observed for 168 h, and mortality was recorded every 12 h. Fish were considered dead when opercular movement had ceased, and no response was observed after gentle mechanical stimulation. No feed was provided during the challenge.

### 2.9 Data processing and analysis

Statistical analyses were performed using SPSS version 26.0, R software version 4.2.0, GraphPad Prism 10, and the Omicsmart online platform.^2^ Normality and homogeneity of variance were assessed using the Shapiro–Wilk and Levene’s tests, respectively. Data that met these assumptions were analyzed using one-way analysis of variance (ANOVA), followed by Tukey’s *post hoc* test. Data that did not meet these assumptions were analyzed using the Kruskal–Wallis test, followed by Dunn’s *post hoc* test with Bonferroni correction.

Spearman’s rank correlation analysis was performed using the cor. Test function in R. For multiple correlation analyses, *p*-values were adjusted using the Benjamini-Hochberg false discovery rate (FDR) method. Microbial alpha diversity and taxonomic abundance were analyzed using R and the Omicsmart online platform. For comparisons involving multiple taxa, *p*-values were adjusted using the Benjamini-Hochberg FDR method. Kaplan–Meier survival curves were generated for the bacterial challenge test, and survival differences were assessed using the log-rank test. Unless otherwise stated, data are presented as mean ± SD, and graphs were generated using GraphPad Prism v.10. For group-level analyses, including growth performance, feed utilization, survival, and challenge-test outcomes, the tank was considered the experimental unit, and analyses were performed using tank-level means, with three replicate tanks per treatment. Differences were considered statistically significant at *p* < 0.05.

## 3 Results

### 3.1 Effects on growth performance indicators and survival rate

Table 3 shows the effects of dietary LP100 supplementation on the growth performance and survival of *S. argus* during the 8-week feeding trial. No mortality occurred in any treatment group before the bacterial challenge, indicating that LP100 supplementation was well tolerated at all the tested concentrations. Compared to the CON and LPL groups, *S. argus* in the LPMH group exhibited significantly enhanced WGR and SGR (*p* < 0.05) and a markedly reduced FCR (*p* < 0.05). Compared with the LPH group, the weight gain rate (WGR) of *S. argus* in the LPMH group was significantly increased (*p* < 0.05), but no significant difference was observed compared to the LPLH group (*p* > 0.05). Compared with the LPLH and LPH groups, there were no significant differences in the SGR of *S. argus* in the LPMH group (*p* > 0.05). In contrast, the feed conversion ratio (FCR) of *S. argus* in the LPMH group was significantly lower than that of the LPL, LPLH, and LPH groups (*p* < 0.05). These results indicate that dietary supplementation with 1.1 × 10^8^ CFU/g LP100 improved growth performance and feed utilization under the present experimental conditions.

**Table 3:** Effects of varying LP 100 concentrations in diet on production performance of *Scatophagus argus.*

Group	CON	LPL	LPLH	LPMH	LPH	η2
Initial weight (*Wi*, g/fish)	20.00 ± 0.50^a^	20.17 ± 0.35^a^	20.30 ± 0.15^a^	19.93 ± 0.05^a^	20.03 ± 0.21^a^	0.23
Final body weight (*W_f_*, g/fish)	94.00 ± 9.17^b^	95.33 ± 8.14^b^	104.4 ± 2.68^ab^	112.6 ± 4.07^a^	101.2 ± 2.75^ab^	0.65
Weight gain rate (WGR, %)	369.7 ± 38.7^b^ (273, 465)	372.9 ± 43.1^b^ (266, 480)	414.1 ± 9.46^ab^ (390, 438)	465.1 ± 21.4^a^ (412, 518)	404.9 ± 9.01^b^ (382, 427)	0.69
Specific growth rate (SGR, % day^−1^)	2.758 ± 0.15^b^ (2.39, 3.13)	2.770 ± 0.17^b^ (2.36, 3.18)	2.923 ± 0.033^ab^ (2.84, 3.01)	3.092 ± 0.067^a^ (2.93, 3.26)	2.891 ± 0.032^ab^ (2.81, 2.97)	0.66
Feed conversion ratio (FCR, g/g)	1.379 ± 0.09^a^ (1.15, 1.61)	1.343 ± 0.05^ab^ (1.22, 1.47)	1.252 ± 0.04^b^ (1.16, 1.34)	1.121 ± 0.02^c^ (1.07, 1.17)	1.265 ± 0.04^b^ (1.16, 1.37)	0.80
Survival rate (SR, %)	100.00 ± 0.00^a^	100 ± 0.00^a^	100 ± 0.00^a^	100 ± 0.00^a^	100 ± 0.00^a^	-

Data are presented as mean ± standard deviation (SD), 95% CI are given in parentheses. Partial *η*^2^ represents the effect size. Values with different superscript letters within the same row are significantly different at *p* < 0.05.

### 3.2 Effects of serum biochemical and immune parameters

Table 4 shows the effects of dietary supplementation with different concentrations of LP100 on serum biochemical and immune activity in *S. argus*. Compared with the control group, LZM activity in the serum of fish from the LPL, LPLH, LPMH, and LPH groups was significantly higher (*p* < 0.05), with the LPMH group exhibiting the greatest increase. For complement components, serum levels of C3 and C4 were elevated in all experimental groups. Specifically, serum C3 and C4 levels in the LPLH, LPMH, and LPH groups were significantly higher than those in the control group (*p* < 0.05), displaying a dose-dependent increasing trend, with the LPH group achieving the highest values. Additionally, serum ALP activity was significantly elevated in the LPLH, LPMH, and LPH groups compared with the CON group (*p* < 0.05); however, no significant differences in ALP activity were detected between the LPMH group and the LPLH or LPH groups.

**Table 4:** Effects of different LP100 concentrations in diets on serum biochemical and immune activity of *S. argus.*

Serum biochemical and immunological indicators	CON	LPL	LPLH	LPMH	LPH
LZM (U/L)	4.075 ± 0.204^c^	5.079 ± 0.0556^b^	5.426 ± 0.445^ab^	6.175 ± 0.436^a^	5.872 ± 0.354^ab^
C3 (μg/mL)	81.05 ± 8.80^b^	87.12 ± 5.41^b^	111.5 ± 11.2^a^	115.4 ± 10.9^a^	127.8 ± 6.35^a^
C4 (μg/mL)	103.1 ± 8.37^c^	112.4 ± 7.82^c^	142.2 ± 3.88^b^	152.9 ± 11.6^ab^	165.3 ± 16.1^a^
ALP (IU/L)	133.0 ± 13.5^b^	147.2 ± 9.26^b^	175.6 ± 12.3^a^	190.7 ± 5.70^a^	193.2 ± 10.7^a^

Data are presented as mean ± standard deviation (SD). Values with different superscript letters within the same row are significantly different at *p* < 0.05.

### 3.3 Effects on antioxidant capacity

Table 5 shows the effects of dietary supplementation on hepatic antioxidant parameters in *S. argus*. Compared with the CON group, hepatic SOD activity was significantly higher in all LP100-supplemented groups, with the highest activity observed in the LPMH groups (*p* < 0.05). Hepatic MDA content was significantly lower in the LPMH and LPH groups than in the CON group (*p* < 0.05). However, the levels did not differ significantly between the LPMH and LPH groups (*p* > 0.05). These results indicate that dietary supplementation with 1.1 × 10^8^ CFU/g LP100 was associated with improved hepatic antioxidant status under the present experimental conditions.

**Table 5:** Effects of different LP100 concentrations in diets on liver antioxidant activity of *Scatophagus argus.*

Liver antioxidant indicators	CON	LPL	LPLH	LPMH	LPH
SOD (U/g)	31.35 ± 0.898^c^	42.24 ± 3.37^b^	46.54 ± 3.34^b^	53.512 ± 0.400^a^	30.49 ± 2.70^c^
MDA (nmol/g)	11.90 ± 0.746^a^	11.68 ± 0.673^a^	10.54 ± 0.414^b^	8.201 ± 0.264^c^	8.366 ± 0.381^c^

Data are presented as mean ± standard deviation (SD). Values with different superscript letters within the same row are significantly different at *p* < 0.05.

### 3.4 Intestinal cytokine gene expression

**Figure 1:**
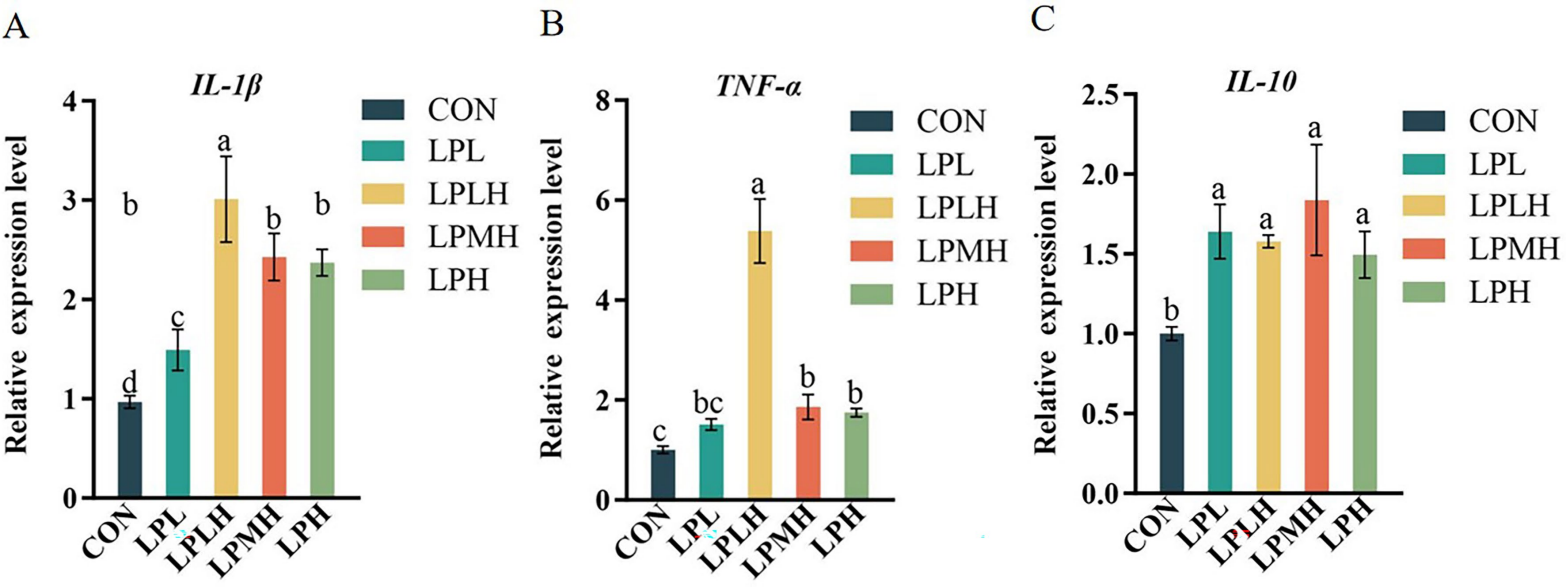
Effects of dietary LP100 supplementation on intestinal cytokine gene expression in *Streptococcus argus*: *IL-1β* **(A)**, *TNF-α* **(B)**, and *IL-10* **(C)**. Data with different letters are significantly different at *p* < 0.05.

Figure 1 shows the effects of dietary LP100 supplementation on intestinal *IL-1β*, *TNF-α*, and *IL-10* expression in *S. argus*. Compared with the CON group, *IL-1β* gene expression was significantly higher in the LPL, LPLH, LPMH, and LPH groups (*p* < 0.05), with the highest expression observed in the LPLH group. No significant differences in *IL-1β* expression were detected among the LPMH and LPH groups (*p* > 0.05). *TNF-α* expression was significantly upregulated in the LPLH, LPMH, and LPH groups compared to the control group (*p* < 0.05). Furthermore, the LPLH group showed the highest expression levels among all treatments, being significantly greater than those in all other groups (*p* < 0.05). *IL-10* expression was significantly higher in all LP100-supplemented groups than in the CON group (*p* < 0.05), with the highest expression observed in the LPMH group. No significant differences in *IL-10* expression were detected among the LP100-supplemented groups (*p* > 0.05).

### 3.5 Gut signaling molecule gene expression

**Figure 2:**
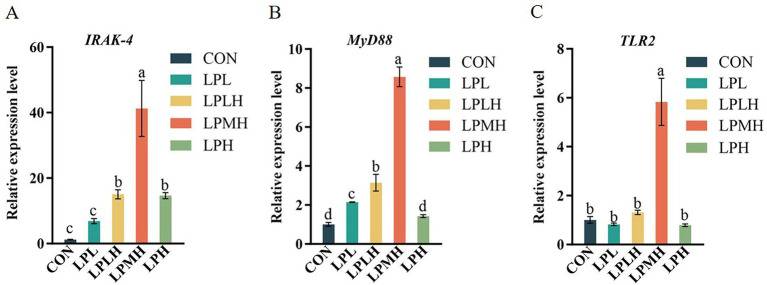
Effects of dietary LP100 supplementation on intestinal immune-related signaling gene expression in *Streptococcus argus*: *IRAK-4* **(A)**, *MyD88* **(B),** and *TLR2* **(C).** Data with different letters are significantly different at *p* < 0.05.

Figure 2 shows the effects of dietary LP100 supplementation on intestinal *IRAK-4*, *MyD8*8, and *TLR2* expression in *S. argus*. Compared with the CON group, *IRAK-4* expression was significantly higher in the LPLH, LPMH, and LPH groups (*p* < 0.05), with the highest expression observed in the LPMH group. *IRAK-4* expression was also significantly higher in the LPMH group than in the LPL, LPLH, and the LPH groups (*p* < 0.05). Compared with the CON group, *MyD88* expression was significantly higher in the LPL, LPLH and LPMH groups (*p* < 0.05), with the highest expression observed in the LPMH group. *TLR2* expression was significantly higher in the LPMH group than in the CON, LPL, LPLH, and LPH groups (*p* < 0.05).

### 3.6 Intestinal morphology

**Figure 3:**
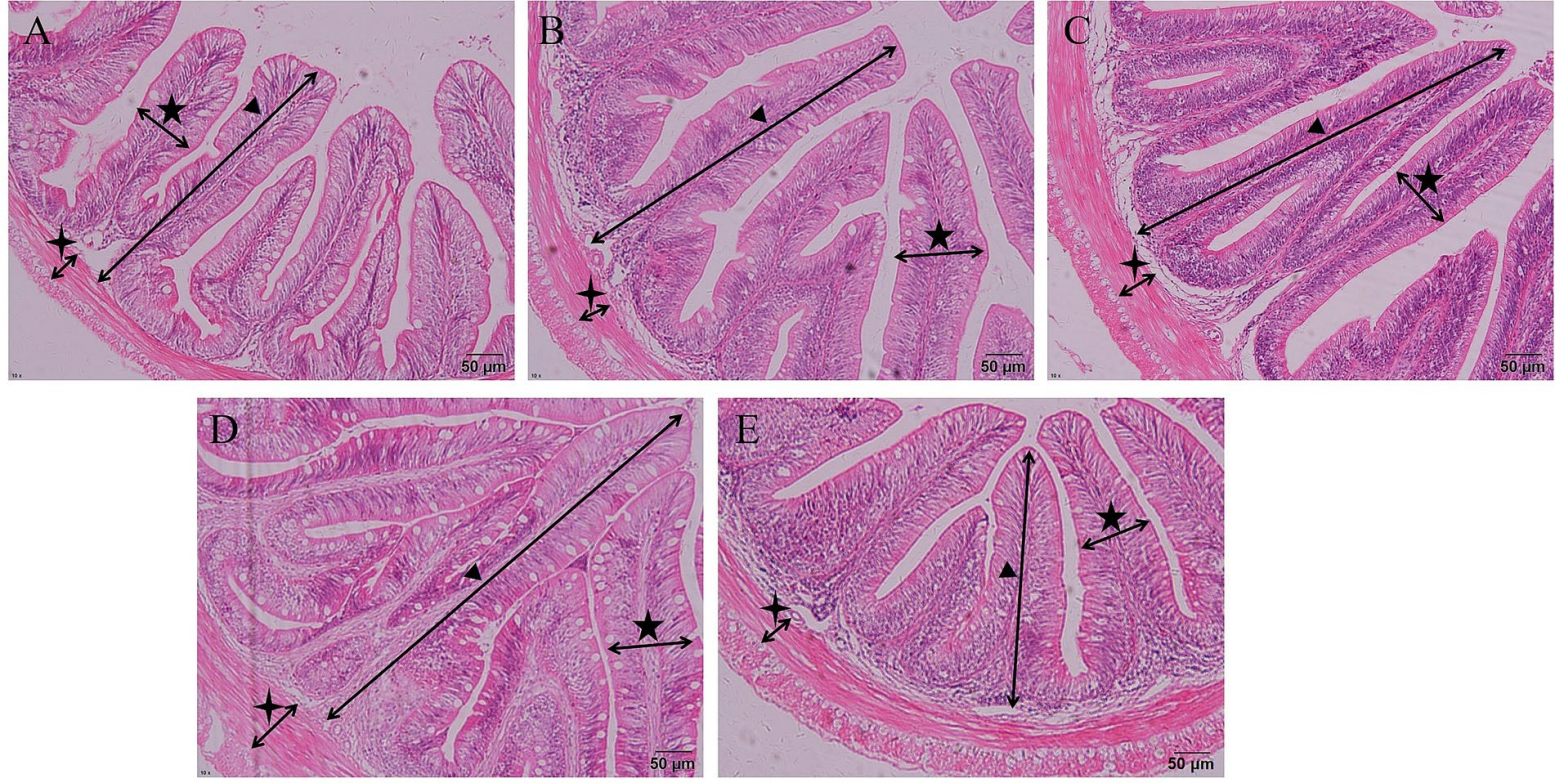
Effects of dietary LP100 supplementation on intestinal histomorphology in *Streptococcus argus* (× 100). **(A)** CON; **(B)** LPL; **(C)** LPLH; **(D)** LPMH; **(E)** LPH. ★ Villus width, ▼ villus length, 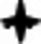 myometrial thickness.

Figure 3, Table 6 show the effects of dietary LP100 supplementation on intestinal villus length (μm), villus width (μm), and muscularis thickness (μm) in *S. argus.* Compared with the CON group, all LP100-supplemented groups had significantly greater villus length (*p* < 0.05). The LPMH group showed the greatest villus length (684.9 ± 64.22 μm), which was significantly higher than that in the LPL, LPLH, and LPH groups (*p* < 0.05). Villus width was also significantly greater in the LPMH group than all other groups (*p* < 0.05), whereas no significant differences were detected among the LPL, LPLH, and LPH groups (*p* > 0.05). A similar pattern was observed for muscularis thickness, which was significantly greater in the LPMH group (108.3 ± 7.798 μm) than in all other groups (*p* < 0.05).

**Table 6:** Histological parameters of the intestinal tissue of *Scatophagus argus* fed with different concentrations of LP100.

Parameter measured	CON	LPL	LPLH	LPMH	LPH
Villus length (μm)	402.4 ± 16.43^d^	490.9 ± 21.49^c^	587.9 ± 6.752^b^	684.9 ± 64.22^a^	448.9 ± 38.16^cd^
Villus width (μm)	100.9 ± 11.39^b^	107.4 ± 9.679^b^	113.5 ± 11.89^b^	128.7 ± 9.990^a^	107.7 ± 7.762^b^
Myometrial thickness (μm)	28.06 ± 8.395^d^	44.17 ± 7.142^c^	62.51 ± 4.722^b^	108.3 ± 7.798^a^	55.18 ± 4.394^b^

Data are presented as mean ± standard deviation (SD). Values with different superscript letters within the same row are significantly different at *p* < 0.05.

### 3.7 Gut microbiota composition

**Figure 4:**
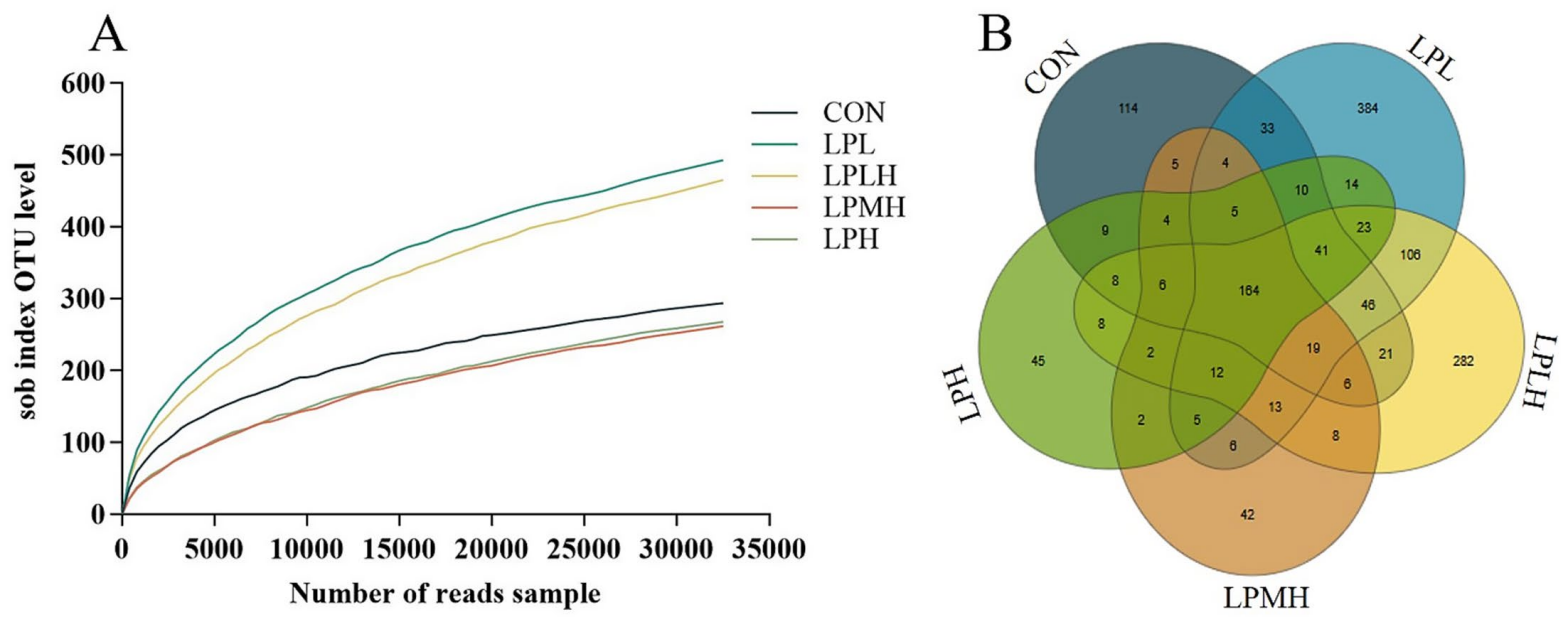
Effects of dietary LP100 supplementation on intestinal microbiota diversity of *Streptococcus argus.* **(A)** Rarefaction curves of gut microbiota. **(B)** Venn diagram of gut microbial operational taxonomic units (OTUs).

Figure 4 shows the effects of dietary LP100 supplementation on gut microbial diversity in *S. argus*. Rarefaction curves approached a plateau across all groups, indicating that the sequencing depth was sufficient to capture most of the microbial diversity in the samples (Figure 4A). No significant differences in OTU counts were detected among the LP100-supplemented groups (*p* > 0.05), suggesting that LP100 supplementation did not significantly alter overall OTU richness under the present experimental conditions. The Venn diagram showed that the CON, LPL, LPLH, LPMH, and LPH groups contained 495, 885, 765, 303, and 358 OTUs, respectively, with 164 OTUs shared across all groups (Figure 4B). The number of group-specific OTUs was 114, 384, 282, 42, and 45 in the CON, LPL, LPLH, LPMH, and LPH groups, respectively. No significant differences in group-specific OTU numbers were detected among groups (*p* > 0.05).

**Figure 5:**
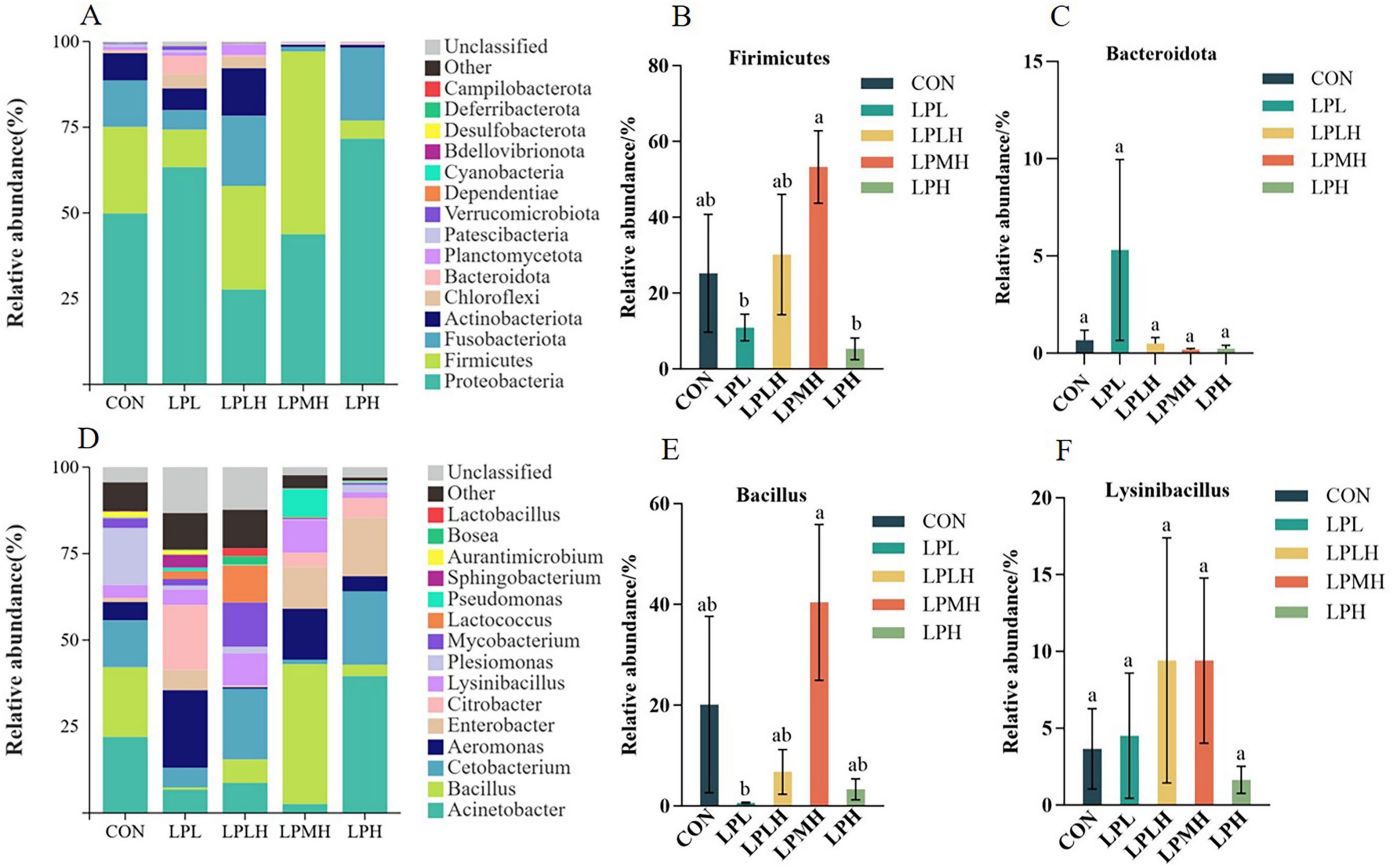
Effects of dietary LP100 supplementation on the relative abundance of intestinal microbiota in *Streptococcus argus*. **(A)** Relative abundance of gut microbiota at the phylum level. **(B)** Relative abundance of Firmicutes. **(C)** Relative abundance of Bacteroidota. **(D)** Relative abundance of gut microbiota at the genus level. **(E)** Relative abundance at genus level of *Bacillus.* **(F)** Relative abundance of *Lysinibacillus*. Data with different letters are significantly different at *p* < 0.05.

Figure 5 shows the effects of dietary LP100 supplementation on the relative abundance of intestinal microbiota in *S. argus*. At the phylum level, the 15 most abundant bacterial phyla were Proteobacteria, Firmicutes, Fusobacteriota, Actinobacteriota, Chloroflexi, Bacteroidota, Planctomycetota, Patescibacteria, Verrucomicrobiota, Dependentiae, Cyanobacteria, Bdellovibrionota, Desulfobacterota, Deferribacterota, and Campilobacterota (Figure 5A). Firmicutes abundance was highest in the LPMH group and was significantly higher in this group than in the LPL and LPH groups (*p* < 0.05; Figure 5B). Bacteroidota abundance did not differ significantly among groups (*p* > 0.05), although the lowest value was observed in the LPMH group (Figure 5C). At the genus level, the 15 most abundant bacterial genera were *Acinetobacter*, *Bacillus, Cetobacterium*, *Aeromonas*, *Enterobacter*, *Citrobacter*, *Lysinibacillus*, *Plesiomonas*, *Mycobacterium*, *Lactococcus*, *Pseudomonas*, *Sphingobacterium*, *Aurantimicrobium*, *Bosea*, and *Lactobacillus* (Figure 5D). *Bacillus* abundance was significantly higher in the LPMH group than in all other groups (*p* < 0.05; Figure 5E). *Lysinibacillus* abundance showed a slight increase in the LPLH and LPMH groups, but the differences among groups were not significant (*p* > 0.05).

### 3.8 Effect of LP100 on the survival rate of fish infected with *S. agalactiae*

**Figure 6:**
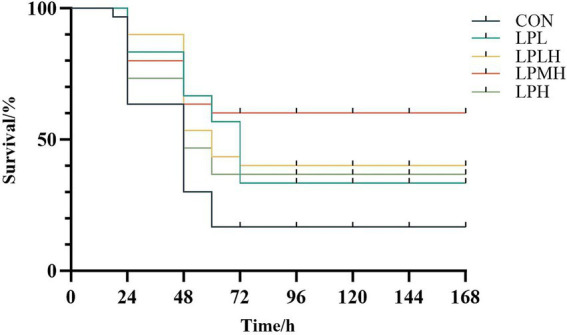
Effects of dietary LP100 supplementation on survival of *Streptococcus argus* after *Streptococcus agalactiae* challenge.

After the 8-week feeding trial, fish from each group were challenged with *S. agalactiae*. In contrast to the feeding trial, in which no mortality was observed, the challenge test resulted in varying degrees of mortality. Figure 6, Table 7 show the survival rates of *S. argus* after *S. agalactiae* challenge. The survival rates of the CON, LPL, LPLH, and LPMH groups were 16.67, 33.33, 40, and 60%, respectively. Survival was significantly higher in the LPL, LPLH, and LPMH groups than in the CON group (*p* < 0.05), with the highest survival observed in the LPMH group. No significant differences were detected between the LPL or LPLH groups and the LPMH group (*p* > 0.05). Kaplan–Meier survival analysis confirmed a significant difference between the LPMH and CON groups (*p* < 0.05).

**Table 7:** Analysis of differences in Kaplan–Meier curves between the different experimental groups using the log-rank test.

Goup	CON	LPL	LPLH	LPMH	LPH
CON	–	*p < 0.05	^*^p < 0.05	*p < 0.05	0.06^ns^
LPL		–	0.71^ns^	0.15^ns^	0.80^ns^
LPLH			–	0.26^ns^	0.57^ns^
LPMH				–	0.09^ns^

## 4 Discussion

Previous studies have shown that *L. plantarum* can improve growth performance and disease resistance in shrimp and several fish species (Amoah et al., 2023; Amenyogbe et al., 2022; Aly et al., 2008). However, probiotic effects are often dose-dependent, and higher supplementation levels do not necessarily provide additional growth benefits (Eissa et al., 2024; Elbahnaswy et al., 2024; Zhou et al., 2022). Excessive supplementation may also increase feed costs without improving production outcomes, whereas insufficient supplementation may provide limited benefit (Ekasari et al., 2023). In this study, four dietary LP100 concentrations were evaluated in *S. argus:* 1.1 × 10^6^, 1.1 × 10^7^, 1.1 × 10^8^, and 1.1 × 10^9^ CFU/g. The LPMH group showed the best growth response, with higher WGR and SGR and significantly lower FCR than other groups. No mortality occurred during the 8-week feeding trial, indicating that LP100 was well tolerated at all tested concentrations. These results suggest that 1.1 × 10^8^ CFU/g LP100 may improve growth performance and feed utilization in *S. argus* under the present experimental conditions.

Probiotics can modulate immune-related responses in aquatic animals. Lysozyme is a widely distributed hydrolase with antibacterial activity and is commonly used as an indicator of innate immune defense in fish (Ferraboschi et al., 2021; Song et al., 2021; Yang and Yan, 2025). Complement C3 and C4 are central components of the complement system and contribute to pathogen recognition and clearance (Chen et al., 2019; Chen W. et al., 2025; Liao et al., 2023). In contrast, ALP should be interpreted more cautiously. In fish, ALP is involved in physiological defense and metabolism, but increased ALP activity is not specific to immune enhancement and may also reflect hepatic or biliary stress (Gobi et al., 2018; Xie et al., 2024). In the present study, the LPMH group had significantly higher serum LZM activity and C3, C4, and ALP levels than the CON group. These results suggest that LP100 at 1.1 × 10^8^ CFU/g may support innate immune and complement-associated defense responses in *S. argus*, mainly as reflected by LZM, C3, and C4. However, the increase in ALP should be interpreted as a broader physiological response rather than as direct evidence of enhanced immunity.

The immune status of fish is closely linked to the regulation of oxidative stress. MDA is a lipid peroxidation product commonly used as an indicator of oxidative damage, whereas SOD is an antioxidant enzyme that catalyzes the removal of superoxide anion radicals (Arafa et al., 2024; Wang et al., 2024). In this study, hepatic SOD activity increased across all LP100-supplemented groups, with the highest in the LPMH group. Hepatic MDA content was significantly lower in the LPMH group than in the CON group. These findings suggest that moderate dietary LP100 supplementation may improve hepatic antioxidant status and reduce oxidative damage in the liver of *S. argus*. Similar growth-promoting effects of dietary *L. plantarum* have been reported in Nile tilapia (*O. niloticus*)(Meachasompop et al., 2025), carp (*Cyprinus carpio*) (Hoseini et al., 2023; Yousefi et al., 2023), and rainbow trout (*Oncorhynchus mykiss*) (Hoseini et al., 2022), although the effective dose varies among host species and probiotic strains. However, oxidative stress markers were measured only in liver tissue. Therefore, these results should not be interpreted as evidence of systemic or intestinal antioxidant effects. Future studies should assess oxidative stress markers in serum, intestine, and other tissues to determine whether LP100 has broader antioxidant effects.

The intestine is an important site of immune defense in fish, and cytokines are the main mediators of intestinal immune responses (Etyemez Büyükdeveci et al., 2023). In this study, dietary LP100 altered the intestinal expression of immune-related cytokines in *S. argus,* including the pro-inflammatory cytokines *IL-1β* and *TNF-α* and the anti-inflammatory cytokine *IL-10*. Interestingly, *TNF-α* peaked in the LPLH rather than the LPMH group, deviating from a typical dose–response pattern. This may be because the moderate dose (LPLH) elicited a strong inflammatory response, while the higher dose (LPMH) simultaneously induced host immunoregulation (e.g., *IL-10* up-regulation), suppressing excessive *TNF-α* production. Although *IL-1β* and *TNF-α* are involved in host defense, excessive or sustained increases in these cytokines can contribute to chronic inflammation, barrier disruption, villus atrophy, and dysbiosis (Coccia et al., 2012; Al-Sadi et al., 2013). Therefore, increased pro-inflammatory cytokine expression should not be interpreted alone as evidence of improved intestinal health.

In the present study, the increase in pro-inflammatory cytokine expression was accompanied by an increase in *IL-10* expression. Histopathological examination of the mid-intestine also showed no evidence of overt intestinal inflammation, such as villus atrophy or epithelial erosion. This pattern suggests that LP100 may have induced a moderate immune-priming response rather than pathological inflammation. Functionally, this immune-related expression pattern was associated with higher disease resistance after *S. agalactiae* challenge. The LPMH group had a significantly higher post-challenge survival rate than the control group (60% vs. 16.7%). Similar regulation of pro- and anti-inflammatory factors has been reported after lactic acid bacterial supplementation in carp (Feng et al., 2019), Nile tilapia (Xia et al., 2018), largemouth bass (Yang et al., 2025), and flounder (Hasan et al., 2018). However, bacterial load quantification was not performed in this study. Thus, whether the observed cytokine profile contributed directly to bacterial clearance remains to be determined.

Toll-like receptors (TLRs) are transmembrane receptors that link innate immune recognition with downstream immune signaling. *TLR2* activates the *MyD88*-dependent pathway and recruits *IRAK-4,* leading to downstream production of inflammatory cytokines (Kim et al., 2007; Wang et al., 2024). In this study, the LPMH diet increased the expression of intestinal *TLR2*, *MyD88*, and *IRAK4* in *S. argus*. This finding suggests that LP100 may modulate the intestinal *TLR2/MyD88* signaling axis. However, TLR-pathway activation in the absence of pathogen challenge does not necessarily indicate improved immune function and may also reflect low-grade inflammatory stress.

The concurrent increase in *IL-10* expression, the absence of obvious histological inflammation, and the higher survival rate after *S. agalactiae* challenge support the interpretation that the LPMH response reflected a potentially controlled immune-readiness state rather than inflammatory injury. One possible mechanism is that LP100 interacts with the intestinal mucosa after colonization, where bacterial cell wall components, such as lipoteichoic acid, may activate TLR2/MyD88 signaling. This could induce a balanced response in which pro-inflammatory cytokines contribute to host defense preparation, while *IL-10* helps limit excessive inflammation and maintain mucosal homeostasis. This interpretation is consistent with the concept of mucosal tolerance and trained immunity in fish (Petit and Wiegertjes, 2016). Similar TLR-pathway responses have been reported after dietary *Enterococcus faecium* supplementation in tilapia (Li et al., 2020), heat-inactivated *B. subtilis* SE5 supplementation in grouper (Yang et al., 2019), and *Aeromonas* SE6 exposure in grouper intestine (Liu et al., 2021). However, LP100 colonization, TLR2 activation, and downstream pathway activity were not directly measured in this study; therefore, this mechanism remains hypothetical.

The intestine is the main site of nutrient digestion and absorption in fish, and intestinal morphology is closely related to growth and physiological condition (Zhang C. et al., 2024). Villus length, villus width, and muscularis thickness are commonly used structural indicators of intestinal condition in aquatic animals (Hien et al., 2021; Wang et al., 2024). Previous studies have shown that probiotics can increase villus height, villus density, and intestinal wall thickness in aquatic species (Cerezuela et al., 2013; Li et al., 2019). A denser and more developed villus structure may increase the absorptive surface area of the intestine and support nutrient uptake.

Similar intestinal morphological changes have been reported after dietary *L. plantarum* supplementation in Japanese eel (*Anguilla japonica*) (Lee et al., 2017), freshwater crayfish (*Cherax cainii*) (Foysal et al., 2021), and *Haliotis discus hannai* (Ke et al., 2025), including increased intestinal wall thickness and villus length. In tilapia, dietary supplementation with *Lactobacillus rhamnosus* increased the height and width of intestinal folds (Sewaka et al., 2019). In the present study, dietary LP100 was associated with greater villus length, villus width, and muscularis thickness in *S. argus*, particularly in the LPMH group. These findings suggest that an appropriate LP100 level may support intestinal structural development and may contribute to nutrient absorption.

However, the present data should be interpreted as morphological evidence rather than direct evidence of improved barrier function. Tight junction proteins, mucus layer thickness, goblet cell density, and intestinal permeability were not assessed. Therefore, the mechanism underlying the LP100-associated morphological changes remains unclear. Future studies should include these functional indicators to determine whether LP100-induced morphological changes improve intestinal barrier integrity.

The gut microbiota is involved in nutrient absorption, immune modulation, and disease resistance in fish (Liu X. et al., 2024; Zhang B. et al., 2024). Consistent with the presence of a core microbiome in fish (Kanika et al., 2024), the predominant microbial taxa in *S. argus* belonged to Firmicutes, Proteobacteria, Fusobacteria, Actinobacteria, Bacteroidota, Verrucomicrobia, and Plantomycetota. Proteobacteria and Firmicutes were the most abundant phyla across all groups, which is consistent with findings in black drum (Yamamoto et al., 2022), turbot (Li et al., 2019), and Atlantic salmon (*Salmo salar*) (Dhanasiri et al., 2023; Gupta et al., 2018). Therefore, the dominance of these two phyla appears to be a baseline feature of the intestinal microbiota in this study rather than an LP100-specific effect.

Compared with the other groups, the LPMH group showed higher Firmicutes abundance and lower Bacteroidota abundance. This finding should be interpreted descriptively, because the functional significance of Firmicutes and Bacteroidota shifts has not been established in *S. argus.* However, Firmicutes include taxa involved in carbohydrate utilization and digestive enzyme production, and their higher abundance in the LPMH group was consistent with the improved WGR, SGR, and FCR observed at this dose (Mang et al., 2024). Proteobacteria abundance remained high across groups, indicating that LP100 did not eliminate Proteobacteria dominance. Because Proteobacteria include both commensal and opportunistic taxa, phylum-level abundance alone is insufficient to determine whether the microbial community was beneficial or dysbiotic. Genus-level changes, therefore provide more useful context for interpreting the LP100-associated microbiota shifts.

At the genus level, LP100 supplementation was associated with changes in both potentially beneficial and opportunistic bacteria. Compared with the control group, the LPMH group showed reduced abundance of potential opportunistic pathogens, including *Acinetobacter* and *Plesiomonas*, and increased abundance of *Bacillus* and *Lysinibacillus*. *Bacillus* species can inhibit pathogens, produce antimicrobial compounds, and enhance mucosal barrier function in aquatic animals (Kuebutornye et al., 2020; Omar et al., 2025). *Lysinibacillus h*as also been associated with improved intestinal morphology, including increased villus height and reduced crypt depth, as well as improved hepatic antioxidant capacity (Fang et al., 2025).

In this study, enrichment of *Bacillus* and *Lysinibacillus* in the LPMH group was associated with greater villus length, villus width, and muscularis thickness, higher SOD activity, and improved post-challenge survival. These parallel changes suggest that the LP100-associated microbial profile may be linked to improved growth, intestinal morphology, immune-related gene expression, and hepatic antioxidant status. However, these associations do not establish causality, and direct functional roles of these taxa were not tested.

Probiotics may produce beneficial metabolites, including organic acids and vitamins, that can serve as supplemental nutrients and support host immune responses against pathogens. For example, dietary *Bacillus* G1-11 enhanced immune responses in hybrid grouper and increased resistance to *Vibrio harveyi* (Zhang et al., 2025). In contrast, *L. plantarum* E2 improved resistance of large yellow croaker (*Larimichthys crocea*) to *Pseudomonas plecoglossicida* (Liu R. et al., 2024). In the present study, LP100 supplementation improved growth performance, increased serum immune-related parameters, altered intestinal immune-related gene expression, and shifted gut microbiota composition. These responses were associated with higher survival following *S. agalactiae* challenge in the LPL, LPLH, and LPMH groups.

After *S. agalactiae* challenge, survival rates in the LPL, LPLH, and LPMH groups were 33.33, 40, and 60%, respectively, and were significantly higher than that in the CON group (16.67%; *p < 0.05*). The LPMH group showed the highest survival rate among the reported groups. However, this result should be interpreted as partial protection under an intraperitoneal challenge model rather than complete protection against natural infection. The LPH group did not show a significant survival benefit compared with the CON group, suggesting that higher LP100 supplementation did not provide additional protection under the present conditions.

The reduced benefit at the highest dose may reflect a dose-effect threshold. Excessive probiotic supplementation may alter microbial balance or immune regulation in a way that does not further improve anti-infective capacity. In contrast, the LPMH dose appeared to produce a more favorable response pattern, including moderate activation of the TLR2/MyD88/IRAK4 pathway, coordinated changes in *IL-1β* and *IL-10* expression, and no obvious histological evidence of intestinal inflammation. The increased abundance of Firmicutes and enrichment of *Bacillus* and *Lysininacillus* may also have contributed to a more favorable intestinal microenvironment. These findings suggest that the protective effect of LP100 may depend on dose-dependent coordination among intestinal microbiota, immune-related gene expression, intestinal morphology, and hepatic antioxidant status.

A limitation of this study is the absence of a concurrent saline-injected negative control in the challenge test. Preliminary data and typical clinical signs, including exophthalmos, abdominal distension, and fin-base hemorrhage, supported infection as the cause of death. However, a saline-injected control would have more clearly distinguished mortality caused by *S. agalactiae* infection from mortality related to injection stress. In addition, the mechanism underlying the LP100-associated survival benefit was not directly tested. Future studies should include a washout period to assess the persistence, tolerance, and reversibility of LP100 effects. Furthermore, bacterial load quantification, histopathological scoring after challenge, cytokine protein measurements, metagenomic and metabolomic analyses, and *in vitro* assays are needed to determine whether LP100 acts through competitive exclusion, quorum-sensing interference, metabolite secretion, or other strain-specific mechanisms.

## 5 Conclusion

Dietary supplementation with LP100 was associated with improved growth performance, hepatic antioxidant status, serum immune-related parameters, intestinal immune-related gene expression, intestinal morphology, gut microbiota composition, and survival after *S. agalactiae* challenge in *S. argus*. Among the four tested concentrations (1.1 × 10^6^, 1.1 × 10^7^, 1.1 × 10^8^, and 1.1 × 10^9^ CFU/g) during the 8-week feeding trial, 1.1 × 10^8^ CFU/g produced the favorable overall response. However, this value should not be interpreted as the definitive optimal dose because only four concentration gradients were tested, the dose intervals were large, and dose–response modeling was not performed. These findings support the potential use of LP100 as a dietary probiotic candidate for *S. argus* aquaculture. Future studies using finer dose gradients, dose–response modeling, bacterial-load quantification, and mechanistic analyses are needed to refine the effective inclusion range and clarify how LP100 affects intestinal barrier function and disease resistance.

## Data Availability

The raw data generated in this study can be found in the NCBI (https://www.ncbi.nlm.nih.gov/), accession PRJNA1479172.

## Footnotes

1
https://github.com/OpenGene/fastp

2
http://www.omicsmart.com
